# A Treatise of Diseases of the Bones

**Published:** 1872-06

**Authors:** 


*A Treatise on Diseases of the Bones.* By THOMAS M. MARKOE, M. D., etc. New York: D. APPLETON & CO. 1872.

Prof. Markoe has given us in this work a very interesting and valuable treatise on diseases of the bones. The work is divided into three parts treating successively diseases of bone, tumors of bone, and malignant diseases of bone. Each of these subjects are treated in a very complete and satisfactory manner and are fully illustrated by cases from the extensive practice by the author.

Each subject is presented in plain and well chosen language, and can not fail to impress the reader that the author has a practical knowledge of what he writes.

The large amount of illustrative cases together with the allusions which are often made to the authorities on the subject of bone pathology give evidence that the author has carefully and in a large number of instances successfully put into practice the result of his investigation and discoveries in diseases of bones. The text is finely illustrated by numerous engravings many of them original, the book is presented to the profession in fine binding and clear type, and is an acquisition of real value in this branch of surgery.

